# Control of amino acid transport coordinates metabolic reprogramming in T-cell malignancy

**DOI:** 10.1038/leu.2017.160

**Published:** 2017-07-11

**Authors:** K M Grzes, M Swamy, J L Hukelmann, E Emslie, L V Sinclair, D A Cantrell

**Affiliations:** 1 Division of Cell Signalling and Immunology, School of Life Sciences, University of Dundee, Dundee, UK; 2 Centre for Gene Regulation and Expression, School of Life Sciences, University of Dundee, Dundee, UK

## Abstract

This study explores the regulation and importance of System L amino acid transport in a murine model of T-cell acute lymphoblastic leukemia (T-ALL) caused by deletion of phosphatase and tensin homolog deleted on chromosome 10 (PTEN). There has been a strong focus on glucose transport in leukemias but the present data show that primary T-ALL cells have increased transport of multiple nutrients. Specifically, increased leucine transport in T-ALL fuels mammalian target of rapamycin complex 1 (mTORC1) activity which then sustains expression of hypoxia inducible factor-1α (HIF1α) and c-Myc; drivers of glucose metabolism in T cells. A key finding is that PTEN deletion and phosphatidylinositol (3,4,5)-trisphosphate (PtdIns(3,4,5)P_3_) accumulation is insufficient to initiate leucine uptake, mTORC1 activity, HIF1α or c-Myc expression in T cells and hence cannot drive T-ALL metabolic reprogramming. Instead, a key regulator for leucine transport in T-ALL is identified as NOTCH. Mass spectrometry based proteomics identifies SLC7A5 as the predominant amino acid transporter in primary PTEN^−/−^ T-ALL cells. Importantly, expression of SLC7A5 is critical for the malignant transformation induced by PTEN deletion. These data reveal the importance of regulated amino acid transport for T-cell malignancies, highlighting how a single amino acid transporter can have a key role.

## Introduction

The proliferation of normal and malignant T lymphocytes is supported by signaling pathways that increase nutrient uptake to meet cellular metabolic demands. Immune activated normal T cells and malignant T cells thus increase glucose uptake and switch to glycolysis to use glucose as a carbon source for their increased biosynthetic demands.^1, 2, 3, 4, 5, 6, 7^ In normal T cells, glucose metabolism is controlled by c-Myc and HIF1 transcription factors which regulate expression of genes encoding glucose transporters and glycolytic enzymes.^4, 8^ The serine/threonine kinase mTORC1 also selectively coordinates glucose transport and glycolysis by controlling the expression of HIF1α.^4, 9^ One important question is whether the metabolic reprogramming of transformed T cells replicates the metabolic reprograming of normal proliferating T cells? In this respect, T-ALL are aggressive tumors of T-cell progenitors caused by mutations in the NOTCH signaling pathway^10^ or mutations/loss of expression of PTEN, a lipid phosphatase with specificity for the 3′ position of PtdIns(3,4,5)P_3_.^11, 12^ T-ALL have high glucose metabolism^5, 6, 7^ and c-Myc,^13, 14, 15^ mTORC1^16, 17, 18^ and HIF1α^19, 20^ are important for their development. However, in contrast to normal T cells, it is not known if there is an mTORC1/HIF regulatory circuit in T-ALL.

One mechanism that coordinates c-Myc and mTORC1 signaling in normal T cells is the control of amino acid uptake.^21^ mTORC1 activity requires sustained leucine and glutamine transport.^22^ Moreover, c-Myc protein has a very short half-life and can only accumulate in T cells exhibiting high levels of amino acid uptake and protein synthesis.^23^ The regulated supply of large neutral amino acids (LNAA) mediated by the System L amino acid transporter SLC7A5 (also known as LAT1) is particularly important in T cells for mTORC1 activity and c-Myc expression.^21^ What about amino acid transport in malignant T cells? Human and mouse malignant T cells express CD98 (SLC3A2),^24, 25^ one subunit of the System L amino acid transporter complex. T-ALL also express *Slc7a5* mRNA and there is evidence that pharmacological blockade of System L transport suppresses leukemia growth.^26^ However there has been no direct analysis of the amino acid transport capacity in primary T-ALL. Accordingly, the present study explores amino acid transport in a mouse model of T-cell leukemia/lymphoma where thymic deletion of the inositol phosphatase PTEN drives rapid T leukemogenesis/lymphomagenesis.^25, 27, 28^ We show that PTEN-null malignant T cells have high membrane transport capacity for multiple nutrients including high System L amino acid transporter activity driven by NOTCH signaling pathways. Moreover, amino acid supply via System L amino acid transporters underpins the metabolic reprogramming controlled by mTORC1, c-Myc and HIF1α in malignant T cells and is critical for the *in vivo* malignant transformation induced by PTEN deletion.

## Materials and methods

### Mice

Mice were maintained in the University of Dundee in compliance with UK Home Office Animals (Scientific Procedures) Act 1986. C57BL/6 *Pten*^fl/fl^*Lck*-Cre, *Hif1a*^fl/fl^*Lck*-Cre, *Pten*^fl/fl^*Hif1a*^fl/fl^*Lck*-Cre and *Pten*^fl/fl^*Slc7a5*^fl/fl^*Lck*-Cre mice were bred and genotyped as described in Supplementary Methods. Experiments were performed using mice between 4 and 6 weeks of age when studying non-transformed PTEN^−/−^ T cells, to ensure the absence of transformed T cells.

### Cell cultures and flow cytometry

F04 and F15 murine PTEN^−/−^ T-ALL cells,^29^ primary murine cytotoxic T cells,^30^ OP9-DL-1 and control OP9 bone marrow stromal cells^31^ were maintained as described previously. Standard flow cytometric protocols were used to monitor surface antigens and intracellular S6 phosphorylated on Ser235 and Ser236.^32^ Details of antibodies used are in Supplementary Methods. Data were acquired on LSR Fortessa or FACSVerse machines (Becton Dickinson, Oxford, UK) and analyzed using FlowJo software (TreeStar, Ashland, OR, USA).

### Nutrient uptakes

Glucose, glutamine and leucine transport were measured using [^3^H]-2-deoxyglucose (1 μCi ml^−1^), [^3^H]-L-glutamine/[^14^C]-L-glutamine or [^3^H]-l-leucine as described previously.^21^ APC-transferrin uptake were performed as described previously.^23^

### Protein detection and mRNA quantitation

Immunoblotting for protein expression and phosphorylation and label free quantitative mass spectrometry protocols for protein quantification were performed as described in Hukelmann *et al*.^9^ and detailed protocols and details of antibodies used are in Supplementary Methods. The mass spectrometry proteomics data have been deposited to the ProteomeXchange Consortium via the PRIDE (1) partner repository with the dataset identifier PXD006209. Details of mRNA purification and protocols for quantitation by RT-PCR are in Supplementary Methods.

### Statistical analyses

Data sets were analyzed using SigmaPlot 12.5 (Systat) or Prism 6.0 (GraphPad). A Shapiro–Wilk test for normality was performed to determine suitable tests for parametric or non-parametric populations. F-tests were performed to determine equal variance of populations, otherwise tests assuming unequal variance were performed. All utilized tests were two-tailed and are stated in the respective figure legends. Multiple comparisons in one-way ANOVA analyses were corrected for using the Holm–Sidak method. Kaplan–Meier survival analyses were performed for the *Pten*^fl/fl^*Lck*-Cre, *Pten*^fl/fl^*Hif1α*^fl/fl^*Lck*-Cre and *Pten*^fl/fl^*Slc7a5*^fl/fl^*Lck*-Cre tumor model.

## Results

### Increased transport of leucine and multiple nutrients in primary PTEN^−/−^ T-ALL cells

**Figure 1:**
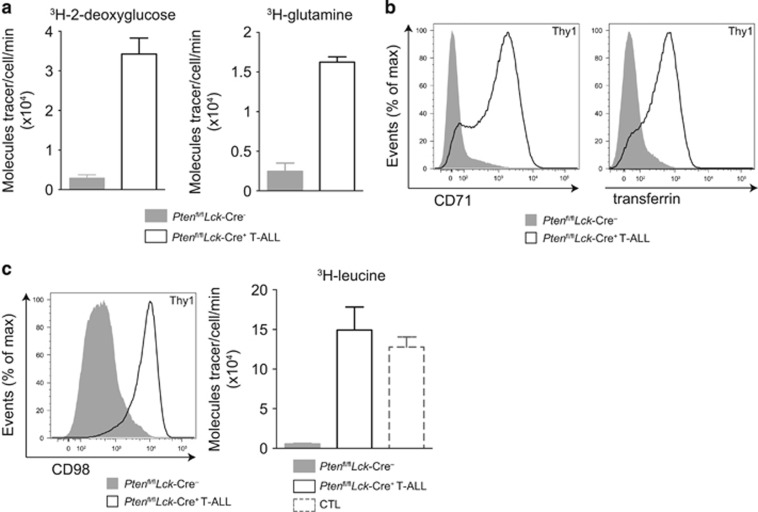
PTEN^−/−^ T-ALL cells upregulate nutrient transport. Thymocytes isolated from *Pten*^fl/fl^*Lck*-Cre^-^ or tumor-bearing *Pten*^fl/fl^*Lck*-Cre^+^ (T-ALL) mice were assayed for (**a**) ^3^H-2-deoxyglucose and ^3^H-glutamine uptake, (**b**) CD71 expression and APC-transferrin uptake and (**c**) CD98 expression and ^3^H-leucine uptake; ^3^H-leucine uptake in cytotoxic T cells (CTL) is shown as a positive control. The glucose analog 2-deoxyglucose was used to indicate glucose uptake. Representative nutrient uptake data shown include technical triplicate values, error bars indicate standard deviation. The data shown are representative of three biological replicates.

Mice with PTEN alleles floxed by loxP Cre excision sequences were backcrossed to *Lck*-Cre transgenic mice that express Cre recombinase selectively in thymic T-cell progenitors. *Pten*^fl/fl^*Lck*-Cre^+^ mice serve as a murine model of T-ALL as mice develop aggressive T-cell lymphomas in the thymus that are fatal at ~8–12 weeks.^25, 28^ Primary *ex vivo* PTEN^−/−^ T-ALL cells can be isolated from *Pten*^fl/fl^*Lck*-Cre^+^ mice for analysis of nutrient transport. Figure 1a shows high rates of glucose and glutamine uptake, respectively, in primary *ex vivo* T-ALL cells isolated from *Pten*^fl/fl^*Lck*-Cre^+^ mice compared with the nutrient uptake of wild-type thymocytes. Primary PTEN^−/−^ T-ALLs also expressed CD71, the transferrin receptor and had high rates of transferrin uptake compared with wild-type thymocytes (Figure 1b). Moreover, PTEN^−/−^ T-ALL cells expressed high levels of CD98, a subunit of System L amino acid transporters. They also had increased uptake of the large neutral amino acid (LNAA) leucine compared with wild-type thymocytes. This was comparable to leucine transport levels of CTL (cytotoxic T cells) (Figure 1c) used as a positive control due to their high rates of amino acid transport.^3, 4, 21, 23^ Two murine PTEN^−/−^ T-ALL cell lines derived from *Pten*^fl/fl^*Lck*-Cre^+^ mice, F04 and F15,^29^ also showed constitutively high transport of multiple nutrients including glucose, glutamine, transferrin and leucine as well as high expression of CD71 and CD98 (Supplementary Figure 1).

### Nutrient transport in T-ALL is not directly driven by PtdIns(3,4,5)P_3_ or AKT

**Figure 2:**
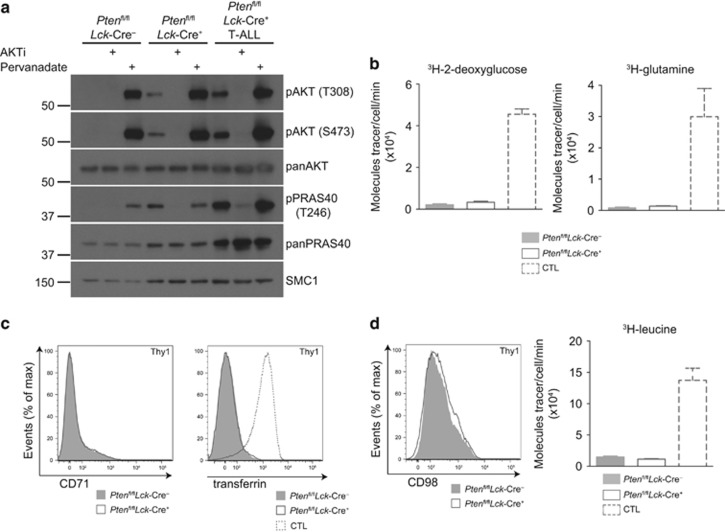
PTEN^−/−^ non-transformed thymocytes have active AKT but do not upregulate nutrient uptake. Thymocytes were isolated from *Pten*^fl/fl^*Lck*-Cre^-^, *Pten*^fl/fl^*Lck*-Cre^+^ and tumor-bearing *Pten*^fl/fl^*Lck*-Cre^+^ (T-ALL) mice. (**a**) Immunoblots for phospho-AKT (T308) and (S473), phospho-PRAS40 (T246) and total AKT, PRAS40 and SMC1. Where indicated, cells were treated with Akti inhibitor (1 μm, 1 h), or pervanadate (100 μm, 10 min) to drive maximum AKT activity. (**b**) ^3^H-2-deoxyglucose and ^3^H-glutamine uptake from thymocytes of indicated genotypes. (**c**) Flow cytometry data of CD71 expression and APC-transferrin uptake. (**d**) CD98 expression and ^3^H-leucine uptake from thymocytes of indicated genotypes. When indicated CTL were used as a positive control. The data shown are representative of (**a**, **c**) 3; (**b**, **d**) 4 biological replicates for each genotype.

In many cells, signaling pathways mediated by PtdIns(3,4,5)P_3_ and the serine/threonine kinase AKT control nutrient transport.^33^ However, in normal effector T cells the regulation of nutrient uptake is phosphatidylinositol-3 kinase (PI3K)/AKT independent.^3, 4^ The *Pten*^fl/fl^*Lck*-Cre^+^ mouse model allows the isolation of PTEN^−/−^ non-transformed thymocytes from young (4–6 week old) mice.^34^ These PTEN^−/−^ non-transformed thymocytes have been well characterized^25, 34^ and are known to be polyclonal and lack the secondary mutations associated with T-ALL. They had high levels of active AKT and increased phosphorylation of the AKT substrate PRAS40 T246 (Figure 2a). The analysis of nutrient uptake in PTEN^−/−^ non-transformed thymocytes allows an assessment of the ability of PtdIns(3,4,5)P_3_ and AKT to drive nutrient transport in T cells. The data show that PTEN^−/−^ non-transformed thymocytes did not increase glucose or glutamine transport (Figure 2b) nor did they express CD71 or increase transferrin uptake (Figure 2c). PTEN^−/−^ non-transformed thymocytes also had very low CD98 expression and leucine transport (Figure 2d). The failure to see increased nutrient transport in PTEN^−/−^ non-transformed thymocytes shows that AKT activation is insufficient for these processes. Thus, the changes in nutrient transport in PTEN^−/−^ T-ALL must be a consequence of the secondary mutations that drive malignant transformation in these cells.^35, 36^

### Expression of c-Myc, HIF1α and mTORC1 activity in PTEN^−/−^ T-ALL

**Figure 3:**
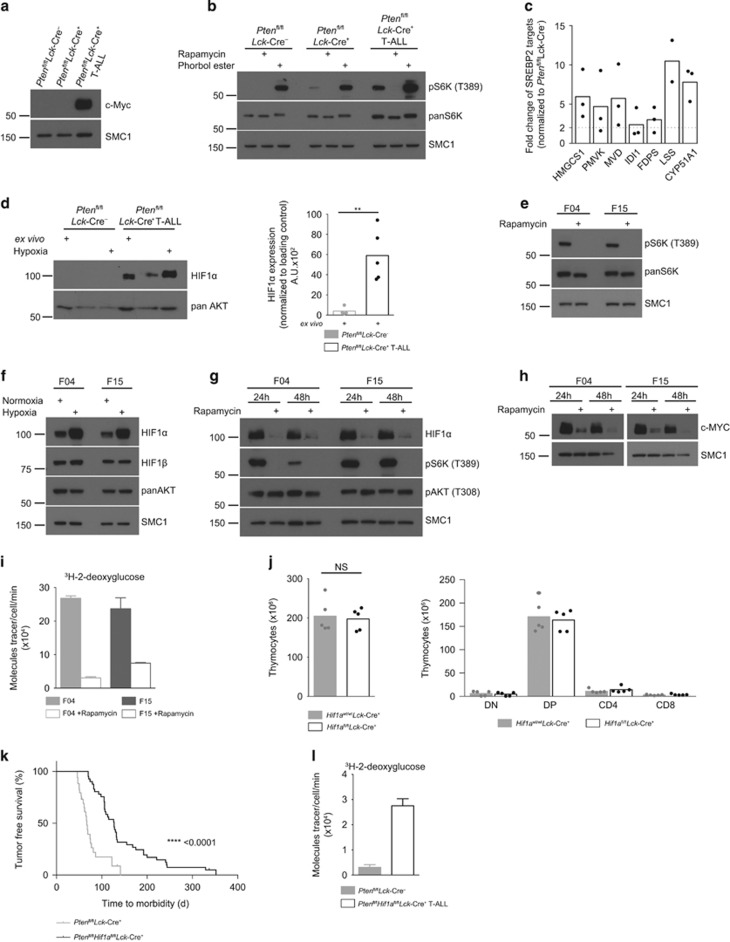
Active mTORC1 controls HIF1α expression that is important for tumorigenesis in PTEN^−/−^ T-ALL. (**a**–**d**) Thymocytes were isolated from *Pten*^fl/fl^*Lck*-Cre^-^, *Pten*^fl/fl^*Lck*-Cre^+^ and tumor-bearing *Pten*^fl/fl^*Lck*-Cre^+^ (T-ALL) mice. Immunoblots show (**a**) c-Myc and SMC1 expression, (**b**) phospho-S6K (T389), S6K and SMC1 expression. Thymocytes were treated as indicated with rapamycin (20 nm, 1 h), or phorbol ester (25 ng ml^−1^, 1 h) as a positive control; (**c**) whole proteome mass spectrometry analysis of thymocytes isolated from three tumor-bearing *Pten*^fl/fl^*Lck*-Cre^+^ (T-ALL) mice compared with thymocytes from three wild-type *Pten*^fl/fl^*Lck*-Cre^-^ mice. Shown is the fold change of estimated copy numbers of SREBP2 targets. (**d**) Immunoblot for HIF1α and SMC1 expression from thymocytes lysed directly after isolation (*ex vivo*) or cultured in 21% (normoxia) or 1% (hypoxia) of O_2_ for 4 h (left panel). Right panel: densitometry analysis (ImageJ) of HIF1α expression in *ex vivo* isolated cells relative to loading control, each point is a biological replicate, *n*=4 for *Pten*^fl/fl^*Lck*-Cre^-^ and *n*=5 for *Pten*^fl/fl^*Lck*-Cre^+^, **P<0.01 (*t*-test). (**e**–**h**) Immunoblot data from T-ALL cell lines F04 and F15 show (**e**) expression of phospho-S6K (T389), total S6K and SMC1 from cells treated with rapamycin (20 nm, 1 h) or untreated; (**f**) shows expression of HIF1α, HIF1β, AKT and SMC1 from cells cultured in 21% (normoxia) or 1% (hypoxia) O_2_ for 4 h and (**g**) expression of HIF1α, phospho-S6K (T389), phospho-AKT (T308) and SMC1 and (**h**) c-MYC and SMC1 from cells +/− rapamycin (20 nM) for indicated times. (**i**) ^3^H-2-deoxyglucose uptake of F04 and F15 cells treated with or without rapamycin (20 nm, 48 h). (**j**) Thymocyte numbers from *Hif1a*^wt/wt^*Lck-*Cre^+^ and *Hif1a*^fl/fl^*Lck-*Cre^+^ mice, each point shown is a biological replicate, *n*=5. Total thymic cell number (left panel); thymocyte progenitor populations (right panel). NS-not significant (*t*-test). (**k**) Kaplan–Meier survival plot comparing kinetics of tumor development in *Pten*^fl/fl^*Lck-*Cre^+^ (*n*=34) mice with *Pten*^fl/fl^*Hif1a*^fl/fl^*Lck-*Cre^+^ (*n*=41) mice. *P*-value was calculated by Log rank (Mantel–Cox) test. (**l**) ^3^H-2-deoxyglucose uptake from *ex vivo* isolated *Pten*^fl/fl^*Hif1a*^fl/fl^*Lck-*Cre^-^ and *Pten*^fl/fl^*Hif1a*^fl/fl^*Lck-*Cre^+^ T-ALL cells. The data shown are representative of (**a**, **b**) 5 and (**k**) 3 biological replicates for each genotype. The data shown in (**e**–**i**) are representative of four independent experiments.

Increases in glucose, glutamine and transferrin uptake in immune activated normal T cells are regulated by c-Myc and mTORC1, the latter via control of expression of HIF1 complexes.^4, 8, 23^ High levels of c-Myc expression are characteristic of T-ALL either as a result of *c-Myc* translocations or NOTCH signaling.^14, 15, 37, 38^ Hence, wild-type and PTEN^−/−^ non-transformed thymocytes had no detectable expression of c-Myc (Figure 3a) and NOTCH1 (Supplementary Figure 2a), whereas c-Myc protein was readily observed in primary PTEN^−/−^ T-ALL cells (Figure 3a). What about mTORC1 activity and HIF1α expression in primary PTEN^−/−^ T-ALL cells? Initially we probed mTORC1 activity by analyzing the phosphorylation of p70S6 kinase 1 (S6K). Figure 3b shows high levels of S6K phosphorylation on T389, the mTORC1 substrate site, in primary *ex vivo* isolated PTEN^−/−^ T-ALL cells. This S6K T389 phosphorylation was lost when cells were treated with the mTORC1 inhibitor, rapamycin. We also assessed mTORC1 activity in PTEN^−/−^ T-ALL cells by quantifying levels of enzymes that control lipid biosynthesis. Their expression is known to be controlled via mTORC1 regulation of the activity of sterol regulatory element binding proteins (SREBPs).^39^ This analysis revealed upregulation of SREBP regulated signaling pathways in PTEN^−/−^ T-ALL cells compared with wild-type thymocytes (Figure 3c). We then analyzed the expression of HIF1α and found that *ex vivo* isolated PTEN^−/−^ T-ALL cells expressed HIF1α which was in stark contrast to wild-type thymocytes where no HIF1α was detected (Figure 3d; Supplementary Figure 2b). Hypoxia promotes the accumulation of HIF1α:^40^ levels of HIF1α in *ex vivo* PTEN^−/−^ T-ALL PTEN^−/−^ T-ALL cells declined in normoxia (21% O_2_) but were increased by hypoxia (1% O_2_) (Figure 3d). In contrast, *ex* *vivo* isolated wild-type and PTEN^−/−^ non-transformed thymocytes did not have high mTORC1 activity (Figure 3b), nor did they express detectable HIF1α or increase HIF1α expression in hypoxia (Supplementary Figures 2b and c) although they did express HIF1β (Supplementary Figure 2d).

To address if HIF1α expression in T-ALL cells is controlled by mTORC1 we switched to experiments with PTEN^−/−^ T-ALL cell lines.^29^ Figure 3e shows that the murine PTEN^−/−^ T-ALL cell lines, F04 and F15, had high levels of mTORC1 activity, as judged by the rapamycin sensitive phosphorylation of S6K T389, and expressed HIF1α in hypoxia and normoxia (Figure 3f). Inhibition of mTORC1 with rapamycin, resulted in a strong decrease in HIF1α expression (Figure 3g). Inhibition of mTORC1 also caused a decrease in expression of c-Myc (Figure 3h). Furthermore, mTORC1 activity was also required to sustain glucose uptake in PTEN^−/−^ T-ALL cells (Figure 3i).

How important is the ability of mTORC1 to control HIF1α expression in PTEN^−/−^ T-ALL cells? In this context, selective deletion of mTORC1 activity in T-cell progenitors extends mouse life span in models of leukemogenesis induced by PTEN deletion;^18^ although cells that have genetically disrupted mTORC1 signaling eventually develop T-cell tumors. *Hif1α*^fl/fl^ *Lck-*Cre^+^ mice had normal numbers and frequencies of thymocyte subsets, indicating that deletion of HIF1α in T-cell progenitors in the thymus does not impair normal T-cell development (Figure 3j). *Pten*^fl/fl^*Lck*-Cre^+^ mice had a median survival of 68 days and only a small percentage of these mice (14%) survived beyond 100 days. In contrast, *Pten*^fl/fl^*Hif1α*^fl/fl^ *Lck-*Cre^+^ mice showed prolonged survival with a median life span of 127 days, although they eventually developed PTEN^−/−^ x HIF1α^−/−^ T-cell tumors (Figure 3k) that had high rates of glucose transport (Figure 3l; Supplementary Figure 3). HIF1α is thus important although not essential for T lymphomagenesis caused by PTEN deletion and HIF1α independent signaling pathways can control glucose transport in T-ALL.

### T-ALL and System L amino acid transport

**Figure 4:**
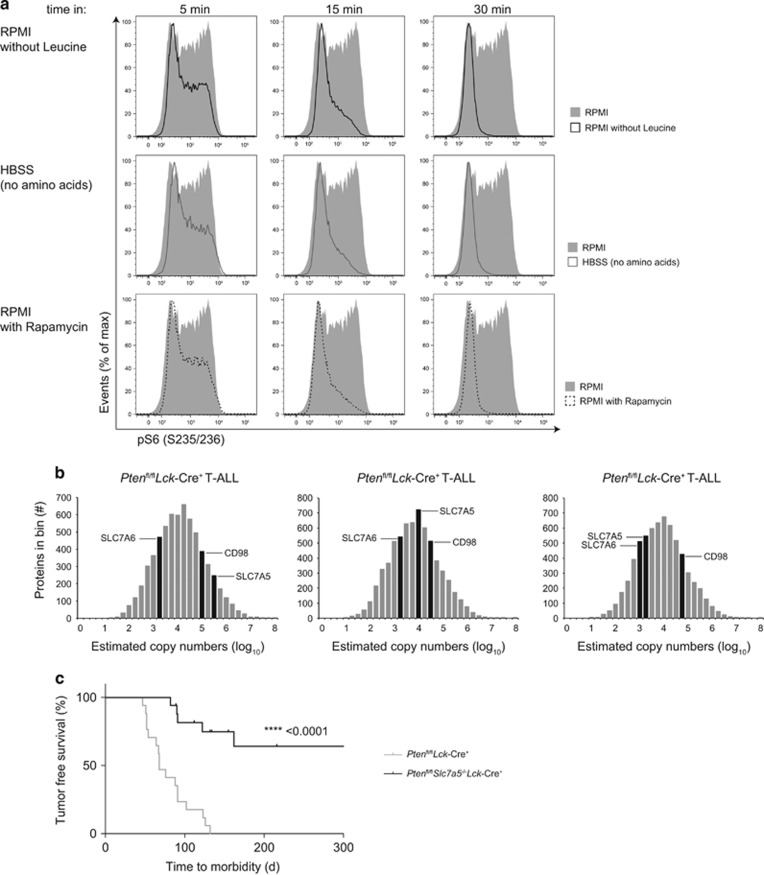
Leucine transporter SLC7A5 is crucial for mTORC1 activity and tumorigenesis in PTEN^−/−^ T-ALL cells. (**a**) Flow cytometry of phospho-S6 (S235/236) expression in murine F04 T-ALL cells maintained in complete RPMI +/− rapamycin (20 nm) or RPMI lacking leucine or HBSS (no amino acids) for indicated times. The data are representative of three independent experiments. (**b**) Histograms showing the distribution of estimated copy numbers of individual proteins as measured by whole proteome mass spectrometry from thymocytes isolated from three tumor-bearing *Pten*^fl/fl^*Lck*-Cre^+^ (T-ALL) mice. The estimated protein copy number of CD98, SLC7A6 and SLC7A5 are indicated. Protein copy number is quantified with the proteome ruler and presented as log-transformed mean values. (**c**) Kaplan–Meier survival plot comparing kinetics of tumor development in *Pten*^fl/fl^*Lck-*Cre^+^ (*n*=18) mice compared with *Pten*^fl/fl^*Slc7a5*^fl/fl^*Lck-*Cre^+^ (*n*=18) mice. *P*-value was calculated by Log rank (Mantel–Cox) test.

One striking observation was the low levels of mTORC1 activity in PTEN^−/−^ non-transformed thymocytes compared with the high mTORC1 activity in malignant PTEN^−/−^ T-ALL cells (Figure 3b). PtdIns(3,4,5)P_3_ accumulation and AKT activation (Figure 2a) were thus insufficient for mTORC1 activition in thymocytes. One key requirement for mTORC1 activity in PTEN^−/−^ T-ALL cells was the sustained transport of leucine (Figure 4a) consistent with the leucine requirement for mTORC1 activity in other cell systems.^21, 41^ Hence, the low mTORC1 activity in PTEN^−/−^ non-transformed thymocytes (Figure 3b) was consistent with the absence of leucine transport in these cells (Figure 2d).

What are the leucine transporters in PTEN^−/−^ T-ALL cells? Leucine is preferentially transported by System L amino acid transporters which are heterodimers consisting of CD98 and either SLC7A5 (LAT1), SLC7A8 (LAT2); SLC7A7 (y^+^LAT1) or SLC7A6 (y^+^LAT2).^42^ Mass spectrometry proteomic analysis of primary *ex vivo* PTEN^−/−^ T-ALL cells from three tumor-bearing *Pten*^fl/fl^ *Lck-*Cre^+^ mice identified that these cells expressed CD98, SLC7A5 and SLC7A6, with SLC7A5 always more abundant than SLC7A6 (Figure 4b). SLC7A5 has a key role in peripheral T cells, controlling mTORC1 activity and c-Myc expression,^21^ known drivers of T-ALL.^13, 43^ In contrast deletion of *Slc7a5* in T-cell progenitors in the thymus does not impair normal T-cell development.^21^ However, SLC7A5 loss dramatically impairs the development of T-cell malignancy: *Pten*^fl/fl^*Slc7a5*^fl/fl^ *Lck-*Cre^+^ mice thus showed prolonged survival (358 days median surivival) compared with the rapid morbidity of *Pten*^fl/fl^*Lck*-Cre^+^ mice (68 day median survival) (Figure 4c). Out of 18 *Pten*^fl/fl^*Slc7a5*^fl/fl^ *Lck-*Cre^+^ mice, only 6 mice developed very late onset PTEN^−/−^ x SLC7A5^−/−^ T-cell tumors (Supplementary Figure 4). SLC7A5 is therefore a dominant System L amino acid transporter in this T-ALL model.

### NOTCH1 regulation of leucine uptake in T-ALL

How do T-ALL control SLC7A5 expression and LNAA transport? We have shown that changes in leucine transport in PTEN^−/−^ T-ALL cells were not a direct consequence of PTEN deletion or AKT activation as they were not seen in PTEN^−/−^ non-transformed thymocytes (Figures 2b–d). We then considered other possible drivers for the changes in leucine transport in T-ALL, and focused on NOTCH. The rationale was that NOTCH activation occurs frequently in human and murine T-ALL^10^ and is known to control mTORC1 activity and expression of c-Myc^15, 44^ and can also drive glucose and glutamine uptake.^45^

**Figure 5:**
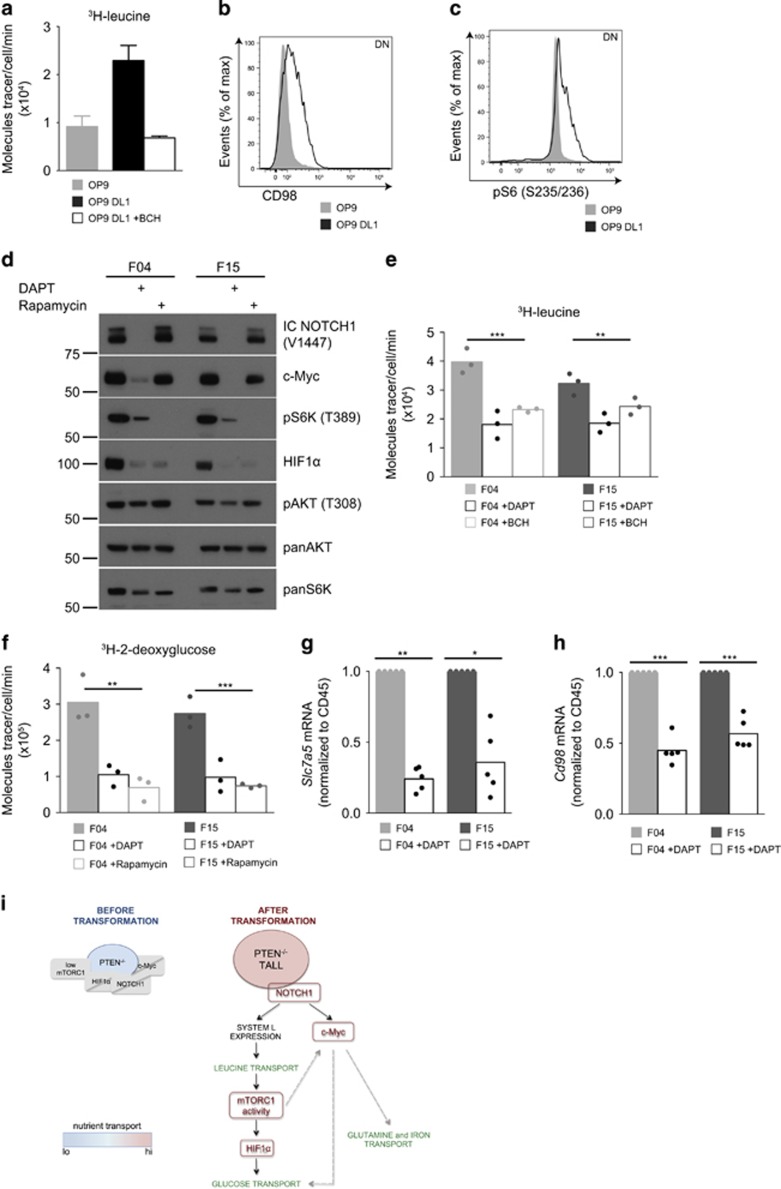
NOTCH signals are essential for maintenance of leucine transport in PTEN^−/−^ T-ALL cells. (**a**–**c**) DN thymocytes isolated from wild-type mice were cultured on OP9 or OP9-DL1 in the presence of IL-7 (5 ng ml^−1^, 48 h) (**a**) ^3^H-leucine uptake +/− System L transport inhibitor BCH (10 mm), (**b**) CD98 expression and (**c**) intracellular phospho-S6 (S235/236) expression. (**d**–**h**) Murine T-ALL cell lines F04 and F15 were treated +/− DAPT (10 μm) or rapamycin (20 nm) for 48 h; (**d**) immunoblot data showing expression of intracellular NOTCH1 (V1744), c-Myc, phospho-S6K (T389), HIF1α, phospho-AKT (T308), AKT and SMC1, (**e**) ^3^H-leucine uptake +/− BCH (10 mm), (**f**) ^3^H-2-deoxyglucose uptake. (**g**) Relative mRNA expression of *Slc7a5* and (**h**) relative mRNA expression of *Cd98.* mRNA levels were normalized to *Cd45*, *P<0.05, **P<0.01, ***P<0.001 (one-way ANOVA). (**i**) Schematic diagram of changes observed in non-transformed thymocytes from *Pten*^fl/fl^*Lck*-Cre^+^ and T-ALL cells from tumor-bearing *Pten*^fl/fl^*Lck*-Cre^+^ mice. PTEN^−/−^ non-transformed thymocytes have low levels of mTORC1 activity and do not express c-Myc, HIF1α or NOTCH1. They also have low levels of nutrient transport. Malignant transformation leads to formation of PTEN^−/−^ T-ALL that have high levels of mTORC1 activity and express HIF1α, NOTCH1 and c-Myc. They also have increased nutrient transport. Active NOTCH1 drives expression of System L transporters and as a consequence leucine transport. Leucine uptake leads to an increase in mTORC1 activity. Active mTORC1 controls expression of HIF1α and glucose uptake in PTEN^−/−^ T-ALL. Additionally, NOTCH1 controls expression of c-Myc, a potential alternative driver for glucose transport.^8^ c-Myc expression is also regulated by mTORC1. Expression of c-Myc can be important for increased transport of glutamine^8^ and iron^23^ in PTEN^−/−^ T-ALL. The data shown are mean from two biological replicates, error bars standard deviation and representative of (**b,c**) three biological replicates, (**d**) four independent experiments. (**e**, **f**) Each point depicts an independent experimental replicate (*n*=3) consisting of the mean of technical triplicates. (**g**, **h**) Each point depicts an independent experimental replicate, *n*=5.

Initial experiments addressed the role of NOTCH in regulating leucine transport directly in wild-type thymocytes. We used the OP9-DL1 system where OP9 cells expressing the NOTCH ligand delta-like 1 (DL1) support the differentiation and self-renewal of T-cell progenitors.^46^ Figure 5a shows that thymocytes maintained in IL-7 on OP9 cells had low levels of leucine transport in contrast to the high levels of leucine uptake in NOTCH-stimulated cells. This leucine uptake was mediated by System L amino acid transporters as evidenced by its sensitivity to the System L competitor BCH (Figure 5a). Leucine uptake was mirrored by the expression of CD98, a subunit of System L amino acid transporter (Figure 5b). Furthermore, only thymocytes that increased leucine uptake showed active mTORC1, as determined by phosphorylation of the S6K substrate, S6 on S235/236 (Figure 5c).

What about the role of NOTCH in established PTEN^−/−^ T-ALL? The T-ALL cell lines F04 and F15 have constitutive NOTCH activity as there is high expression of the NOTCH1 intracellular domain (IC NOTCH1) which is released by NOTCH1 gamma-secretase cleavage between G1743 and V1744 (Figure 5d). The gamma-secretase inhibitor, DAPT, blocked the accumulation of the IC NOTCH1 (V1744) and caused loss of NOTCH transcriptional activity as judged by the loss of expression of c-Myc (Figure 5d). F04 and F15 cells also had high rates of leucine transport that could be partially blocked by NOTCH1 inhibition (Figure 5e). DAPT treated F04 and F15 cells also lost mTORC1 activity, HIF1α expression and glucose transport capacity (Figures 5d and f, respectively). NOTCH inhibition in F04 and F15 cells also decreased expression of *Slc7a5* (Figure 5g) and *Cd98* (Figure 5h). NOTCH signals thus sustain expression of System L amino acid transporters and LNAA transport in PTEN^−/−^ T-ALL cells.

## Discussion

The importance of understanding metabolic checkpoints in malignant cells is now clear. Much work in leukemias has focused on important changes in glucose metabolism but other nutrients such as amino acids and iron are equally important.^26, 47^ The present data highlight how primary PTEN^−/−^ T-ALL cells switch to high rates of transport of multiple nutrients and one discovery is that primary *ex vivo* T-ALL have high System L amino acid transport capacity. Moreover, the regulated transport of LNAA via the amino acid transporter SLC7A5 is key for T-cell malignancy. Amino acid transport via System L transporters is important for protein synthesis but also supplies leucine which is essential to sustain mTORC1 activity in T-ALL cells. mTORC1 is able to control T-ALL metabolism by controlling expression of two key transcription factors c-Myc and HIF1α. The importance of c-Myc for T-cell leukemia is well established.^13^ The present data show that HIF1α is also expressed in primary T-ALL cells and is critical for T-ALL development induced by PTEN deletion. In this respect, the impact of HIF1α deletion on the development of the T-cell tumors following thymic deletion of PTEN phenocopies the impact of losing mTORC1 activity.^18^ One role for HIF1α is to regulate the transcription of genes encoding glucose transporters and glycolytic enzymes. It was noteworthy that when *Pten*^fl/fl^*Hif1α*^fl/fl^ *Lck-*Cre^+^ mice did eventually develop tumors, they had high rates of glucose transport. Therefore, HIF1α independent signaling pathways can mediate glucose metabolism in T-ALL. One alternative regulator is c-Myc which controls the transcription of genes encoding glucose transporters and enzymes controlling glycolysis and glutaminolysis in T cells.^8^ Another key role for c-Myc is to control glucose flux through the hexosamine biosynthetic pathway which is also essential for the T lymphoma/leukemias driven by PTEN deletion.^45^ The importance of c-Myc and mTORC1 controlled pathways for malignant T cells would be one reason that an amino acid transporter such as SLC7A5 would also be important. c-Myc expression and mTORC1 activity in T cells are thus highly dependent on the regulated supply of amino acids via System L amino acid transporters.^21, 23^ mTORC1 and c-Myc hence act as signaling hubs that connect amino acid transport to multiple metabolic processes.

Loss and inactivation of PTEN and elevated PtdIns(3,4,5)_3_ and AKT activity is a common feature of T-ALL malignancy.^12^ In many cells PtdIns(3,4,5)P_3_/AKT signaling has a role to control the activity of mTORC1.^48^ However, we show that PTEN loss and AKT activation alone is not sufficient to activate mTORC1 in T cells. The molecular basis for the inability of PTEN deletion alone to drive mTORC1 activity is that PTEN deletion alone is not sufficient to induce the transport of nutrients such as glucose, glutamine and leucine that are essential for mTORC1 activity. In the context of System L transporter expression, leucine transport and mTORC1 activity in T-ALL cells, the present study identifies NOTCH as a key driver. The engagement of T cells with NOTCH ligands is thus sufficient to induce leucine transport in T cells. Moreover the loss of NOTCH signaling in established T-ALL cells results in loss of leucine transporters and a failure to sustain mTORC1 activity. Previous studies have shown the importance of NOTCH control of c-Myc.^14, 15^ The NOTCH control of amino acid transport shown herein reveals a mechanism that allows NOTCH to co-ordinate the c-Myc and also mTORC1 controlled metabolic pathways that are essential for T-cell tumors (summary Figure 5i). These data reinforce previous ideas that pharmacological blockade of System L transport would be a valuable strategy to supress leukemia growth.^26^
