# Supplementary material for: Control of amino acid transport coordinates metabolic reprogramming in T-cell malignancy
Source: Leukemia. 2017 Jul 11;31(12):2771–9. doi: 10.1038/leu.2017.160 (PMC5729345; doi:10.1038/leu.2017.160)
Supplement: Supplementary Information [file leu2017160x1.docx]

**Supplemental Material**

**Supplemental Methods**

**Mice**

Mice were maintained in the University of Dundee in compliance with UK Home Office Animals (Scientific Procedures) Act 1986. *Pten*^fl/fl^*Lck*-Cre mice were generated as described previously (25,27,28). *Hif1a*^fl/fl^ mice were purchased from The Jackson Laboratory and crossed with mice expressing Cre recombinase under the control of the proximal p56lck promoter (*Lck*-Cre). *Pten*^fl/fl^*Hif1a*^fl/fl^*Lck*-Cre and *Pten*^fl/fl^*Slc7a5*^fl/fl^*Lck*-Cre mice were generated by crossing mice with floxed *Pten* alleles with floxed *Hif1a* alleles or floxed *Slc7a5* alleles and mice expressing Cre recombinase under the control of the proximal p56lck promoter (*Lck*-Cre). Experiments were performed using mice between 4 and 6 weeks of age when studying non-transformed PTEN^-/-^ T cells, to ensure the absence of transformed T cells. All mice were on a C57BL/6 genetic background and were used between 4 and 24 weeks old. Wild type mice used as controls were either WT or WT-Cre^+^ or Cre recombinase-negative with various *Pten*, *Hif1a* and *Slc7a5* floxed alleles.

**PCR Primers used for genotyping:**

*Pten*^fl/fl^ primers:

LoxP Forward 5′-GGTGCTGGTGTCCAAAATGT-3′,

loxP Reverse 5′-GGGCAGTACTGGAAAGATGG-3′,

Reverse 5’-ATAGCTATTAGTGTCTTAACCTGCCC-3’ which generated products of 280 base pairs (*Pten*^fl^), 230 base pairs (wild-type *Pten*) and 380 base pairs (*Pten* deletion).

*Hif1a*^fl/fl^ primers:

LoxP Forward 5′-GCCTTACCTAGTAAAGCAAG-3′,

loxP Reverse 5′-GCAAAGAATCTTGGTGTTAC-3′,

Reverse 5’-GCGCTGCTGGAAACAATCACAAGAA-3’ which generated products of 350 base pairs (*Hif1a*^fl^), 290 base pairs (wild-type *Hif1a*) and 450 base pairs (*Hif1a* deletion).

*Slc7a5*^fl/fl^ primers:

LoxP Forward 5′-TCGGCAACACCCATCCCAGTATCC-3′,

Forward 5′-GGCTCCTGGACTTATCTTGACCAA-3′,

Reverse 5’-GTGGTGCTTTGCTGAAGGCAGGG-3’ which generated products of 359 base pairs (*Slc7a5*^fl^), 271 base pairs (wild-type *Slc7a5*) and 253 base pairs (*Slc7a5* deletion).

Where indicated cells were treated with: mTORC1 inhibitor rapamycin (20 nM, Calbiochem), AKT inhibitor VIII, Akti (1 μM, Calbiochem), NOTCH1 inhibitor DAPT (10 μM), System L inhibitor BCH (2-aminobicyclo-(2,2,1)-heptane-2-carboxylic acid; 10 mM, Sigma), protein tyrosine phosphatase inhibitor pervanadate (100 μM, Sigma), S6K activator phorbol ester (phorbol 12,13-dibutyrate, 25ng/ml, Sigma).

**Cell cultures**

Murine PTEN^-/-^ T-ALL cell lines were a gift from Dr Hergen Spits and were tested for PTEN deletion (Immunoblotting); they were tested mycoplasma negative (LONZA MycoAlert mycoplasma detection kit (#LT07-318). Cells were maintained in IMDM supplemented with 100 U/ml penicillin, 1 mg/ml streptomycin and 10% (vol/vol) heat-inactivated FBS. Primary T cells were activated to generate cytotoxic T lymphocytes (CTL) as described previously (30). OP9-DL-1 and control OP9 bone marrow stromal cells (31) were maintained in alpha-MEM supplemented with 50 μM 2-mercaptoethanol, 100 U/ml penicillin, 100 μg/ml streptomycin and 20% (vol/vol) heat-inactivated FBS. MACS purified DN thymocytes were cultured on OP9 or OP9-DL1 cells for the times indicated in the figure legends. IL-7 was added at 5 ng ml^−1^.

**Flow cytometry and cell sorting**

For cell surface staining, fluorochrome-conjugated antibodies were used to detect (clone indicated in brackets): CD4 (RM4-5), CD8 (53-6.7), CD71 (C2F2), CD98 (RC388) from BD Pharmingen; Thy1 (HIS51) from eBioscience; APC-transferrin (T23366) from Invitrogen. Antibodies were diluted 1:200 unless otherwise indicated, and 1 × 10^6^ cells were stained in 100 μl. Live cells were gated according to their forward scatter and side scatter.

Intracellular staining of phosphorylated S6 at Ser235 and Ser236 was performed as described previously (32), Alexa647-conjugated secondary goat antibody to rabbit was used (111-607-003, 1:5000, Jackson Immunoresearch).

Data were acquired on LSR Fortessa or FACSVerse machines (Becton Dickinson) and analyzed using FlowJo software (TreeStar).

**Nutrient uptake measurements**

Briefly, nutrient uptake was carried out using 0.5–1 × 10^6^ cells resuspended in 0.4 ml uptake medium, layered over oil. After 4 min uptake period, cells were spun through the oil layer to terminate uptake, cell pellets were resuspended in 1mM NaOH and radiocactivity measured by scintillation counter. Glucose, glutamine and leucine uptakes were measured individually in glucose-free RPMI (Life Technologies) containing [^3^H]-2-deoxyglucose (1 μCi ml^−1^), and in HBSS with Ca^2+^ and Mg^2+^ (Life Technologies) containing [^3^H]-L-glutamine (1 μCi ml^−1^) or [^3^H]-L-leucine (1 μCi ml^−1^). In Suppl.Fig.1a glucose and glutamine uptake were measured simultaneously in PBS with Ca^2+^ and Mg^2+^ (Life Technologies) containing [^3^H]-2-deoxyglucose (1 μCi ml^−1^), and [^14^C]-L-glutamine (1 μCi ml^−1^). Uptakes were assayed as described previously (21). The glucose analogue 2-deoxyglucose was used to indicate glucose uptake. Representative nutrient uptake data shown include technical triplicate values, error bars indicate standard deviation.

**Immunoblotting**

*Ex vivo* isolated thymocytes were rested for 30 min in DMEM supplemented with 100 U/ml penicillin, 1 mg/ml streptomycin and 10% (vol/vol) heat-inactivated FBS before the stimulation (unless indicated otherwise), proteins were extracted and standard immunoblotting protocols were used (4). Blots were probed with antibodies recognizing, dilutions and clone/catalog numbers in brackets: pT308 AKT (1:1000, #4056), pS473 AKT (1:1000, #4058), AKT (1:1000, 9272), T246 PRAS40 (1:1000, #13175), PRAS40 (1:1000, #2691), c-Myc (1:1000, #9402), pT389 S6K (1:1000, #9239), S6K (1:1000, #9202), V1744 IC NOTCH1 (1:1000, #4147) from Cell Signaling Technology; HIF1α (1:500, #241809) from R&D Systems; HIF1β (1:1000, #sc-8076) from Santa Cruz Biotechnology; SMC1 (1:5000, A300-055A, Bethyl). Cells were lysed at 4 x 10^7^/ml of *ex vivo* thymocytes and 2 x 10^7^/ml from PTEN^-/-^ T-ALL and CTL.

**Label free quantitative mass spectrometry**

3 biological replicates of *ex vivo* isolated PTEN^-/-^ TALL were isolated and dead cells removed (Miltynei, Biotec) and processed as described previously (9). Briefly, cells were washed twice with ice cold HBSS and 2 x 10^7^ cells were lysed in 0.2 ml of urea lysis buffer (8 M urea, 100 mM Tris-HCl, pH 8.0 and protease and phosphatase inhibitors. Protein concentration was determined by BCA assay (Pierce) and lysates were incubated with DTT (working concentration of 10 mM, Sigma) at 30°C for 30 min, followed by incubation with iodoacetamide (final concentration of 50 mM) at 21°C for 45 min in dark. Next, lysates were digested with lysyl endopeptidase (Wako) at 50:1 ratio (protein/lysyl endopeptidase) at 30°C overnight followed by digestion with trypsin (Promega) at 50:1 ratio at 30°C for 8 h. Resulting peptides were desalted using C18 Sep-Pak cartridges, eluted and reduced to dryness in a vacuum concentrator. Samples were then separated using strong anion exchange chromatography using Dionex RSLCnano HPLC (Thermo Fisher), desalted using tC18 Sep-Pak 96 well plate and analyzed by Velos Orbitrap mass spectrometer (Thermo Fisher). The data were processed, searched and quantified using the MaxQuant software package version 1.4.1.2 as described previously (49), using the default settings and employing the mouse Uniprot database from October 2013 and the contaminants database supplied by MaxQuant. Further downstream analysis was performed using the proteomic ruler in Perseus software (50) to calculate the copy numbers of each protein and Microsoft Excel.

**Quantitative real-time PCR**

RNA was purified with the RNeasy RNA purification Mini Kit (Qiagen) and RNA was reverse-transcribed with the qScript cDNA synthesis kit (Quanta). Quantitative PCR was performed in triplicates in 384 well plate using SYBR Premix Ex Taq II (2X) (TaKaRa) in C1000 Thermal Cycler (Bio-Rad Laboratorories). mRNA levels were calculated based on averaged CT values and normalized to *Cd45* mRNA levels.

**Quantitative PCR primers**

*Slc7a5*: Forward 5′-CTGGATCGAGCTGCTCATC-3′,

Reverse 5′-GTTCACAGCTGTGAGGAGC-3′;

*Cd98*: Forward 5′-GAGGACAGGCTTTTGATTGC-3′

Reverse 5′-ATTCAGTACGCTCCCCAGTG-3′.

**Statistical analyses**

Data sets were analyzed using SigmaPlot 12.5 (Systat) or Prism 6.0 (GraphPad). A Shapiro-Wilk test for normality was performed to determine suitable tests for parametric or non-parametric populations. F-tests were performed to determine equal variance of populations, otherwise tests assuming unequal variance were performed. All utilized tests were two-tailed and are stated in the respective figure legends. Multiple comparisons in one-way ANOVA analyses were corrected for using the Holm-Sidak method. Kaplan-Meier survival analyses were performed for the *Pten*^fl/fl^*Lck*-Cre, *Pten*^fl/fl^*Hif1aα*^fl/fl^*Lck*-Cre and *Pten*^fl/fl^*Slc7a5*^fl/fl^*Lck*-Cre tumor model.

**Supplemental Figure legends**

**Supplemental Figure 1. PTEN^-/-^ F04 and F15 cells have high levels of nutrient transport**

The data show nutrient uptake of murine PTEN^-/-^ F04 and F15 cell lines; **(a)** ^3^H-2-deoxyglucose and ^14^C-glutamine uptake, **(b)** CD71 expression and APC-transferrin uptake, **(c)** CD98 expression and ^3^H-leucine uptake. The data shown are representative of 3 independent experiments. The glucose analogue 2-deoxyglucose was used to indicate glucose uptake. Representative radiolabeled nutrient uptake data shown include technical triplicate values, error bars indicate standard deviation.

**Supplemental Figure 2. Expression of HIF1α and HIF1β in thymocytes from *Pten*^fl/fl^*Lck-*Cre^-^, *Pten*^fl/fl^*Lck-*Cre^+^ and TALL cells from *Pten*^fl/fl^*Lck-*Cre^+^ tumor bearing mice.**

Immunoblotting analysis of thymocytes of indicated genotypes showing **(a)** IC NOTCH1 (V1744), c-Myc and SMC1 expression. The data are representative for 3 biological replicates. **(b,c)** HIF1α and SMC1 or panAKT expression from thymocytes lysed directly after isolation (*ex vivo*) or cultured under 21% (normoxia) or 1% (hypoxia) of O_2_ for 4h in 4 separate experiments. CTL were used as a positive control. **(d)** The data show HIF1β and SMC1 expression from thymocytes lysed directly after isolation or cultured under 21% (normoxia) or 1% (hypoxia) of O_2_ for 4h. The data are representative for 3 biological replicates.

**Supplemental Figure 3. Genotype verification from *Pten*^fl/fl^*Hif1a*^fl/fl^*Lck-*Cre^+^ tumor**

Representative PCR analysis of genomic DNA from thymocytes from *Pten*^fl/fl^*Hif1a*^fl/fl^*Lck-*Cre^-^ and *Pten*^fl/fl^*Hif1a*^fl/fl^*Lck-*Cre^+^ tumor bearing mice. The products of PCR amplification of loxP-flanked (FL), wild-type (WT) and deleted (Del) *Pten* and *Hif1a* alleles are indicated

**Supplemental Figure 4. Genotype verification from *Pten*^fl/fl^*Slc7a5*^fl/fl^*Lck-*Cre^+^ tumor**

Representative PCR analysis of genomic DNA isolated from thymocytes from *Pten*^fl/fl^*Slc7a5*^fl/wt^*Lck-*Cre^-^ and *Pten*^fl/fl^*Slc7a5*^fl/fl^*Lck-*Cre^+^ tumor bearing mice. The products of PCR amplification of loxP-flanked (FL), wild-type (WT) and deleted (Del) *Slc7a5* and *Pten* alleles are indicated.

**References**

49. Cox J, Mann M. MaxQuant enables high peptide identification rates, individualized p.p.b.-range mass accuracies and proteome-wide protein quantification. Nat Biotechnol. 2008 Dec;26(12):1367–72.

50. Wisniewski JR, Hein MY, Cox J, Mann M. A “proteomic ruler” for protein copy number and concentration estimation without spike-in standards. Mol Cell Proteomics. 2014 Dec;13(12):3497–506.
