# Supplementary figures and images for: Control of amino acid transport coordinates metabolic reprogramming in T-cell malignancy
Source: Leukemia. 2017 Jul 11;31(12):2771–9. doi: 10.1038/leu.2017.160 (PMC5729345; doi:10.1038/leu.2017.160)

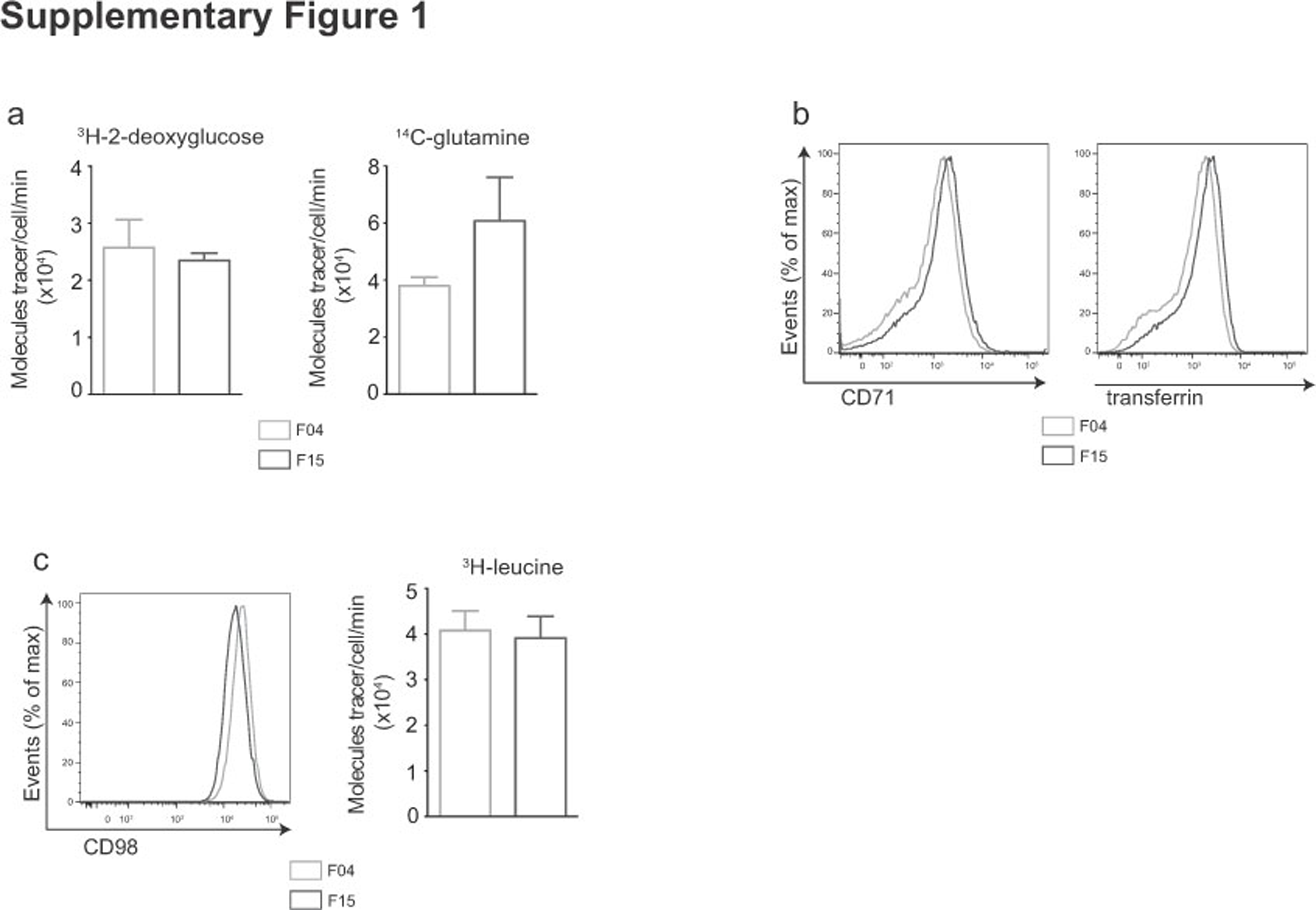

Supplement: Supplementary Figure 1 [file leu2017160x2.tif]

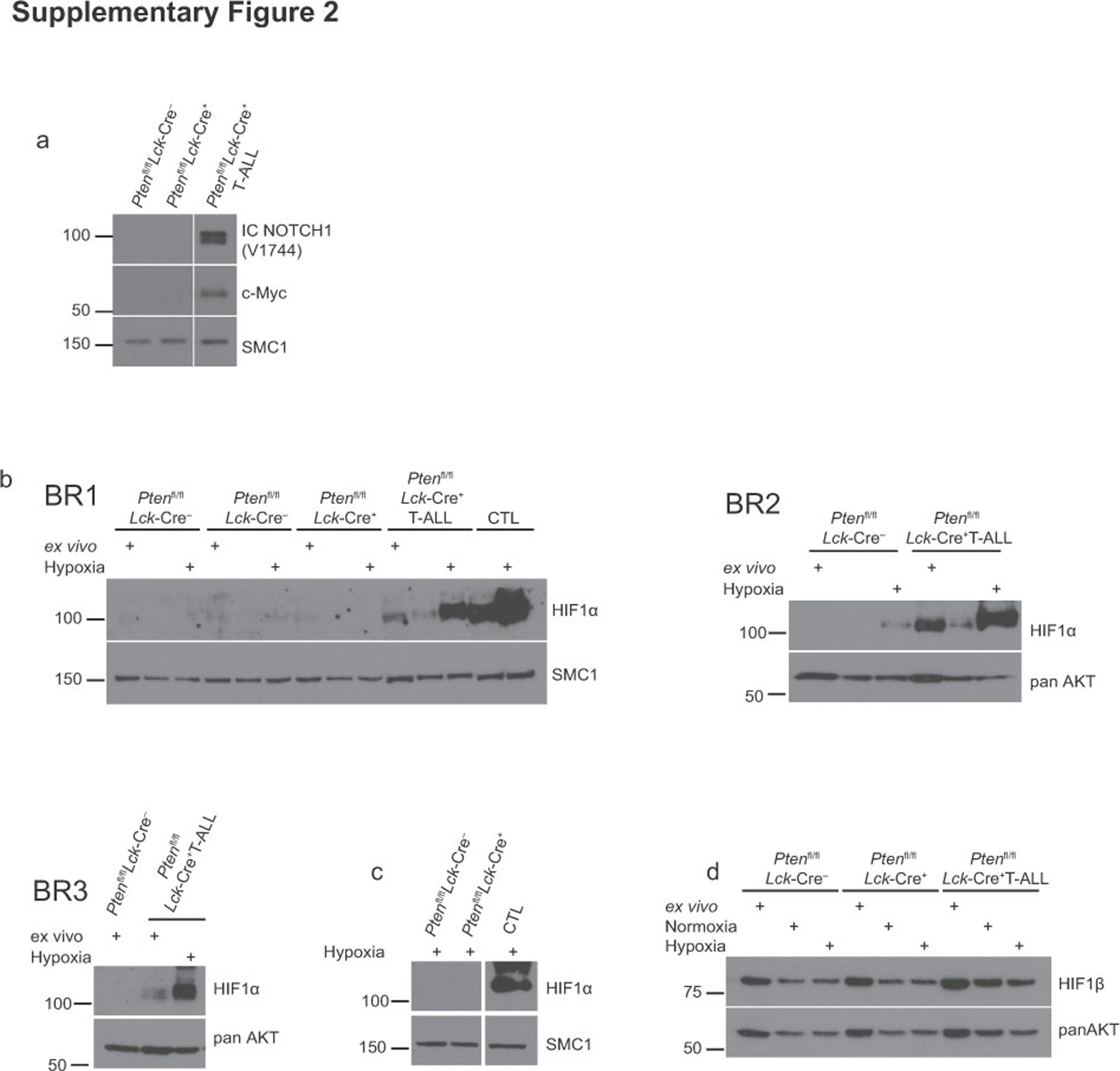

Supplement: Supplementary Figure 2 [file leu2017160x3.tif]

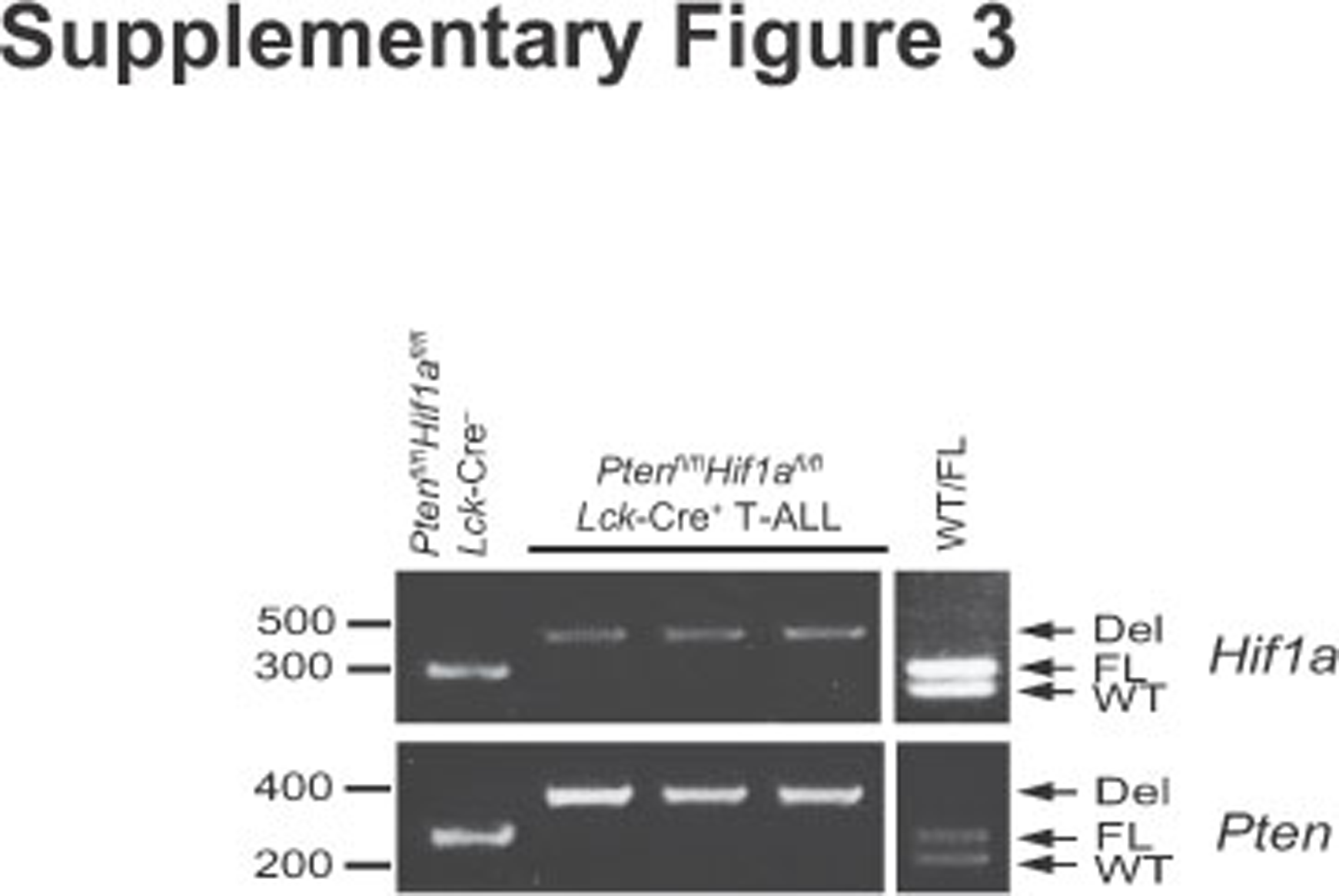

Supplement: Supplementary Figure 3 [file leu2017160x4.tif]

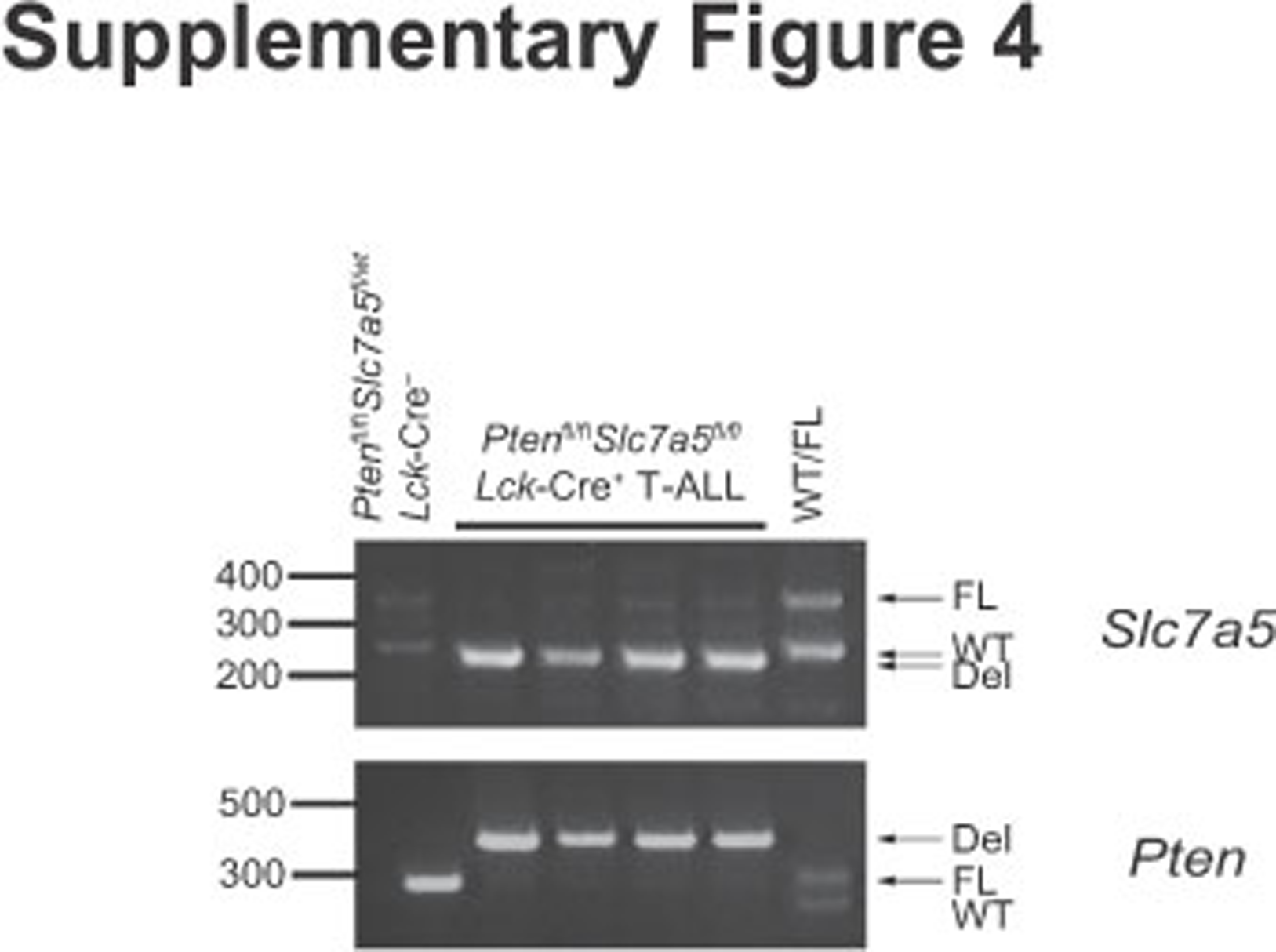

Supplement: Supplementary Figure 4 [file leu2017160x5.tif]
